# Heterologous mRNA/MVA delivering trimeric-RBD as effective vaccination regimen against SARS-CoV-2: COVARNA Consortium

**DOI:** 10.1080/22221751.2024.2387906

**Published:** 2024-08-01

**Authors:** Laura Marcos-Villar, Beatriz Perdiguero, María López-Bravo, Carmen Zamora, Laura Sin, Enrique Álvarez, Carlos Óscar S. Sorzano, Pedro J. Sánchez-Cordón, José M. Casasnovas, David Astorgano, Juan García-Arriaza, Shubaash Anthiya, Mireya L. Borrajo, Gustavo Lou, Belén Cuesta, Lorenzo Franceschini, Josep L. Gelpí, Kris Thielemans, Marta Sisteré-Oró, Andreas Meyerhans, Felipe García, Ignasi Esteban, Núria López-Bigas, Montserrat Plana, María J. Alonso, Mariano Esteban, Carmen Elena Gómez

**Affiliations:** a Department of Molecular and Cellular Biology, Centro Nacional de Biotecnología (CNB), Consejo Superior de Investigaciones Científicas (CSIC), Madrid, Spain; b Centro de Investigación Biomédica en Red de Enfermedades Infecciosas (CIBERINFEC), Instituto de Salud Carlos III (ISCIII), Madrid, Spain; c Department of Microbial Biotechnology, CNB, CSIC, Madrid, Spain; d Biocomputing Unit and Computational Genomics, CNB, CSIC, Madrid, Spain; e Veterinary Pathology Department, Centro de Investigación en Sanidad Animal (CISA), Instituto Nacional de Investigación y Tecnología Agraria y Alimentaria (INIA), CSIC, Madrid, Spain; f Department of Macromolecular Structures, CNB, CSIC, Madrid, Spain; g Center for Research in Molecular Medicine and Chronic Diseases (CiMUS), Universidade de Santiago de Compostela, Santiago de Compostela, Spain; h Laboratory for Molecular and Cellular Therapy, Department of Biomedical Sciences, Vrije Universiteit Brussel, Brussels, Belgium; i Barcelona Supercomputing Center (BSC), Barcelona, Spain; j Department of Biochemistry and Molecular Biomedicine, University of Barcelona (UB), Barcelona, Spain; k Infection Biology Laboratory, Department of Medicine and Life Sciences, University Pompeu Fabra, Barcelona, Spain; l Institució Catalana de Recerca i Estudis Avançats (ICREA), Barcelona, Spain; m Infectious Diseases Department, Hospital Clínic, UB, Barcelona, Spain; n Institut d’Investigacions Biomèdiques August Pi i Sunyer (IDIBAPS), Hospital Clínic, UB, Barcelona, Spain; o Institute for Research in Biomedicine (IRB), Barcelona, Spain; p Centro de Investigación Biomédica en Red de Cáncer (CIBERONC), ISCIII, Madrid, Spain; q Instituto de Investigaciones Sanitarias (IDIS), Santiago de Compostela, Spain

**Keywords:** SARS-CoV-2 vaccine, trimeric-RBD, nanocarriers, mRNA/MVA regimen, vaccine protection

## Abstract

Despite the high efficiency of current SARS-CoV-2 mRNA vaccines in reducing COVID-19 morbidity and mortality, waning immunity and the emergence of resistant variants underscore the need for novel vaccination strategies. This study explores a heterologous mRNA/Modified Vaccinia virus Ankara (MVA) prime/boost regimen employing a trimeric form of the receptor binding domain (RBD) of the SARS-CoV-2 spike (S) protein compared to a homologous MVA/MVA regimen. In C57BL/6 mice, the RBD was delivered during priming via an mRNA vector encapsulated in nanoemulsions (NE) or lipid nanoparticles (LNP), followed by a booster with a replication-deficient MVA-based recombinant virus (MVA-RBD). This heterologous mRNA/MVA regimen elicited strong anti-RBD binding and neutralizing antibodies (BAbs and NAbs) against both the ancestral SARS-CoV-2 strain and different variants of concern (VoCs). Additionally, this protocol induced robust and polyfunctional RBD-specific CD4 and CD8 T cell responses, particularly in animals primed with mLNP-RBD. In K18-hACE2 transgenic mice, the LNP-RBD/MVA combination provided complete protection from morbidity and mortality following a live SARS-CoV-2 challenge compared with the partial protection observed with mNE-RBD/MVA or MVA/MVA regimens. Although the mNE-RBD/MVA regimen only protects half of the animals, it was able to induce antibodies with Fc-mediated effector functions besides NAbs. Moreover, viral replication and viral load in the respiratory tract were markedly reduced and decreased pro-inflammatory cytokine levels were observed. These results support the efficacy of heterologous mRNA/MVA vaccine combinations over homologous MVA/MVA regimen, using alternative nanocarriers that circumvent intellectual property restrictions of current mRNA vaccine formulations.

## GRAPHICAL ABSTRACT

**Figure:**
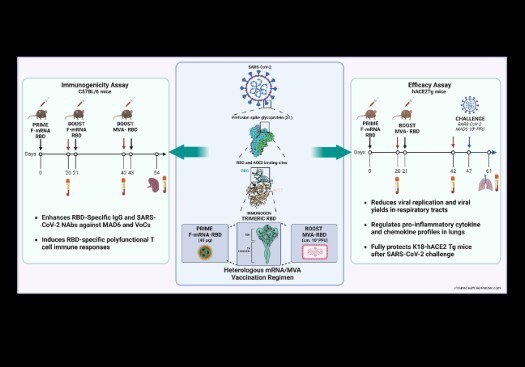

## Introduction

Vaccines have played a critical role in combating COVID-19, significantly reducing severe illness, hospitalizations and mortality. Among the most effective and widely used are mRNA-based vaccines, although other platforms, including viral vector and protein subunit vaccines, have also gained widespread global use [1]. These vaccines target the SARS-CoV-2 S protein, crucial for viral entry. However, the ongoing evolution of the virus, especially the emergence of variants with multiple mutations in the S protein, has challenged the efficacy of current vaccines, highlighting the need for novel strategies to address immune escape and durability of protection [2–4].

The immune response to COVID-19 involves NAbs and cellular cytotoxicity. The RBD of SARS-CoV-2 is a key target for NAbs and vaccine development [5]. It has been demonstrated that vaccine effectiveness correlates with its ability to neutralize SARS-CoV-2 variants, with higher NAbs levels providing better protection [6]. Different studies have shown that targeting the RBD, rather than the full-length S protein, can elicit higher levels of cross-reactive NAbs, offering broad protection against SARS-CoV-2 VoCs [7–11]. Beyond neutralization, Fc-mediated effector functions such as antibody-dependent cellular cytotoxicity (ADCC) could enhance immune protection by enabling natural killer (NK) cells to recognize and eliminate infected cells, complementing the action of NAbs and providing a crucial defense against SARS-CoV-2 [12,13].

Recent advances in vaccine technology have explored MVA-based vectors expressing modified forms of the SARS-CoV-2 S protein. This approach has shown promise in preclinical models, inhibiting SARS-CoV-2 replication using either systemic or mucosal administration [14–21]. MVA-based vaccines, particularly as booster doses in heterologous immunization strategies, have demonstrated their capability to induce specific humoral and cellular immune responses against different pathogens [22–25]. These findings pave the way for using MVA-based vectors in primary vaccination and to enhance immunity in individuals previously immunized with different vaccine types, contributing to more robust and flexible vaccination strategies against COVID-19.

We previously described the design, immunogenicity and efficacy of an mRNA vector expressing a soluble trimeric-RBD form of the Wuhan strain encapsulated in different formulations including NE, nanocapsules (NC) and LNP in preclinical models [26]. To extend these findings, in this study we aim to evaluate the immunogenicity and protective efficacy of a heterologous mRNA/MVA vaccination regimen compared to a homologous MVA/MVA schedule in mice. This approach could offer a promising alternative for human vaccination programs, potentially overcoming the challenges posed by the evolving landscape of SARS-CoV-2.

## Materials and methods

### Cells

HeLa cells (ATCC-CCL-2), DF-1 cells (ATCC-CRL-12203) and Vero-E6 cells (ATCC-CRL-1586) were grown in Dulbecco’s Modified Eagle’s Medium (DMEM; Sigma-Aldrich, St. Louis, MO, USA) supplemented with 2 mM L-glutamine (Sigma-Aldrich), 100 U/mL penicillin / 100 µg/mL streptomycin (Sigma-Aldrich), 0.1 mM non-essential amino acids (Sigma-Aldrich), 0.5 μg/mL amphotericin B (Fungizone; Gibco-Thermo Fisher, Waltham, MA, USA) and 10% heat-inactivated fetal bovine serum (FBS; Sigma-Aldrich). Cell lines were maintained at 37°C in a humidified air, 5% CO_2_ atmosphere.

### Viruses

The poxviruses used in this work included: MVA-WT, provided by Prof. Dr. Gerd Sutter (Ludwig-Maximilians-University of Munich, Munich, Germany), MVA-RBD, expressing a soluble trimeric-RBD form (described below), and MVA-RBDmono, expressing a soluble monomeric-RBD form both from the Wuhan strain. Poxviruses were grown in DF-1 cells and purified through two 36% (w/v) sucrose cushions. The virus titers were determined by immunostaining plaque assay in monolayers of DF-1 cells, as previously described [27].

SARS-CoV-2 strain MAD6 (kindly provided by Prof. Luis Enjuanes and Dr. José M. Honrubia, CNB-CSIC, Madrid, Spain) is a virus isolated from a nasopharyngeal swab from a 69-year-old male COVID-19 patient from Hospital 12 de Octubre in Madrid, Spain, as previously reported [28]. The SARS-CoV-2 VoCs Delta (B.1.617) (SARS-CoV-2, Human, 2021, Germany ex India, 20A/452R) and Omicron: BA.1 (B.1.1.529) (hCoV-19/Belgium/rega-20174/2021, EPI_ISL_6794907), BQ.1.1 (EPI_ISL_15653663) and XBB.1.5 (EPI_ISL_16939528) were kindly provided through Dr. Juan García-Arriaza (CNB-CSIC, Madrid, Spain) as previously reported [26].

### mRNA vectors

The mRNA-RBD encodes the RBD domain (aa: 330–532) from the ancestral Wuhan strain of SARS-CoV-2 modified by the inclusion of a foldon trimerization domain derived from T4 fibritin. This modification was designed to stabilize the trimeric-RBD form, which is how it is naturally located on the virus surface, and to enhance its immunogenicity [7,11,29,30].

Synthesis, production and manufacturing of synthetic mRNA-RBD were performed at Prof. Thielemans’s Lab (Vrije Universiteit Brussel, Brussels, Belgium) as previously described [25]. To confirm the trimeric conformation of RBD expressed from mRNA, 5 μg of naked mRNA-RBD were transfected into HeLa cells grown in 24-well plates using Lipofectamine 2000 (Invitrogen, Carlsbad, CA, USA) and the expression of RBD was analyzed by Western-blotting (WB). As observed in Supplementary Figure 1A, trimeric-RBD is detected in the cellular pellets and supernatants of transfected cells at both 8 and 24 h post-transfection under non-reducing conditions, while monomeric-RBD is detected under reducing conditions mainly in the cellular pellet at 8 h post-transfection and in the supernatant after 24 h of transfection.

Purified mRNA-RBD was encapsulated in three nanoemulsions (NE-1, NE-2 and mNE) and two lipid nanoparticles (mLNP and LNP-1) by solvent-displacement technique, consisting of the controlled mixing of an ethanol phase containing an appropriate amount of lipid components with an aqueous phase containing the mRNA [25]. An organic phase was prepared by dissolving different lipids in ethanol. For NE-1, organic phase consisted on DOTAP (1,2-dioleoyl-3-trimethylammonium propane, chloride salt, from Avanti Polar Lipids, Alabaster, AL, USA), DOPE (1,2-dioleoyl-sn-glycero-3-phosphoethanolamine, from Avanti Polar Lipids), Captex8000NF (tricaprylic acid, from ABITEC Corporation, Columbus, OH, USA) and Tween 80 (Merck Millipore, Burlington, MA, USA). The organic phase of NE-2 contained DOTAP, DOPE, Vitamin E (D,L-α-tocopherol, from BASF, Mannheim, Germany) and Tween 80. An optimized version of NE-1, named mNE, was prepared by adding C12-200 (Alnylam Pharmaceuticals, Cambridge, MA, USA) to the organic phase (already consisting of DOTAP, DOPE, Captex8000 NF and Tween 80) to enhance the transfection efficiency of the NE. For mLNP, the organic phase consisted of C12-200, DOPE, cholesterol (SyntheCholTM USP/NF. Ph.Eur, JP, Sigma-Aldrich SAFC) and DMG-PEG ((R)-methoxy-polyethyleneglycol-2000-carbamoyl-di-O-myristyl-sn-glyceride, gifted by Alnylam Pharmaceuticals). Finally, the organic phase for LNP-1 contained MC3 (D-Lin-MC3-DMA, from TargetMol Chemicals, Boston, MA, USA), DSPC (1,2-distearoyl-sn-glycero-3-phosphocholine, from Avanti Polar Lipids), cholesterol and Tween 80. The resulting organic solutions (containing the appropriate amount of the lipids) were mixed with an aqueous solution (mRNA in citrate buffer, pH 4) by simultaneous injection in a microfluidic system. Formulations were characterized by size, polydispersity and zeta-potential (Zetasizer Nano ZS, Malvern Instruments, UK). Final mRNA concentration and RNA encapsulation efficiency were estimated using Quant-iT RiboGreen RNA assay kit (Invitrogen), following manufacturer’s instructions, and agarose gel assay. These data are summarized in Supplementary Figure 1B.

### Construction and in vitro characterization of MVA-RBD recombinant virus

For the generation of MVA-RBD recombinant virus, the RBD domain (aa: 330–532) construct was codon-optimized for Vaccinia virus usage, synthesized by GeneArt (Thermo Fisher Scientific, Waltham, MA, USA) and subcloned into the plasmid transfer vector pHA, previously described [31], via *BamHI/NotI*. The resulting plasmid pHA-RBD (7407 bp) was checked by PCR and DNA sequence analysis (Macrogen, Seoul, South Korea).

The MVA-RBD recombinant virus, expressing the soluble trimeric-RBD from Wuhan strain, was generated by homologous recombination using the pHA-RBD vector, which directs the insertion of *RBD* gene into the viral HA locus (*A56R* gene) of MVA-WT as previously reported [24,32]. To determine the identity and purity of MVA-RBD viral preparation, DNA was extracted from DF-1 cells infected with MVA-WT or MVA-RBD at 5 plaque-forming units (PFUs)/cell as previously described [32]. Primers HA-MVA and HA2-CAR spanning HA flanking regions were used for the analysis of the HA locus by PCR. The amplification reactions were performed with Phusion High-Fidelity DNA polymerase (BioLabs, Ipswich, MA, USA). To evaluate the correct expression of trimeric-RBD protein, HeLa cells grown in 24-well plates were infected with MVA-RBD at 3 PFUs/cell. At different times post-infection, cellular pellets and supernatants were obtained and analyzed by WB using a rabbit polyclonal anti-RBD antibody (1:1000; GeneTex GTX135385, Irvine, CA, USA). The subcellular localization of RBD protein was assessed by confocal microscopy in HeLa cells infected with MVA-RBD at 1 PFU/cell. After 16 h, cells were treated with fluorescent wheat germ agglutinin conjugated with Alexa Fluor 555 (WGA 555), fixed, permeabilized and stained with a rabbit polyclonal anti-RBD antibody (1:200; GeneTex GTX135385). Goat anti-rabbit-Alexa Fluor 488 was used as secondary antibody (1:500; Life Technologies, Waltham, MA, USA). Leica STELLARIS 5 microscope and the specialized software LasX (Leica Microsystems, Wetzlar, Germany) were used for the acquisition of optical sections of the cells and for image recording and processing, respectively [33]. The stability of MVA-RBD recombinant virus was analyzed by WB of 19 isolated virus plaques obtained after 7 serial infection passages in DF-1 cells at 0.01 PFUs/cell, as previously described [32,33]. The genetic identity of the background MVA before and after the low multiplicity of infection (MOI) passages was determined by the analysis of the HA locus by PCR.

### Size exclusion chromatography (SEC)

Size exclusion chromatography was performed to determine the size of the trimeric-RBD protein expressed by the recombinant MVA-RBD vector. For this, HeLa cells were infected at 3 PFUs/cell with MVA-RBD or MVA-RBDmono expressing the monomeric form of RBD as control and, after 24 h, supernatants were clarified by centrifugation for 20 min at 3000 rpm followed by filtration, concentration to about 0.5 mL and run through a Superdex 200 (10/300) column (Cytiva, Marlborough, MA, USA) with PBS buffer. Fractions were collected (0.5 mL) to determine the elution volume of the RBD by WB under non-reducing and non-denaturing conditions. Subsequently, the intensity of the bands corresponding to the different RBD proteins was determined using Image Lab 6.1 (BIO-RAD, Madrid, Spain) to create the elution profile of each analyzed sample. To estimate the approximate RBD size in the SEC, several size exclusion markers (SIGMA) were run in the same column under the same conditions.

#### Mouse immunizations

To characterize the SARS-CoV-2-specific immune response elicited by the different heterologous mRNA-RBD/MVA-RBD immunization regimens*,* five groups of female C57BL/6JOlaHsd mice (*n* = 5), purchased from Envigo Laboratories (Sant Feliu de Codines, Barcelona, Spain) and housed in the animal facility of CNB-CSIC, were immunized at days 0 and 21 with 40 μg of mRNA-RBD (G1: NE-1-RBD, G2: NE-2-RBD, G3: mLNP-RBD or G4: LNP-1-RBD) or mRNA-control (G5: LNP-1-LUC) by intramuscular (i.m.) route. Three weeks later (day 43), mice were immunized with 1 × 10^7^ PFUs of MVA-RBD (G1 to G4) or MVA-WT (G5 control group) by the same route. At 11 days post-MVA boost (day 54), animals were sacrificed, and spleens were processed for the analysis of the SARS-CoV-2 RBD-specific T cell immune response by intracellular cytokine staining (ICS) assay as previously described [24]. Blood was collected by submandibular bleeding at days 20 and 42 and at sacrifice (d54) to determine SARS-CoV-2-specific binding and neutralizing antibodies [33].

For the efficacy study, six groups of female K18-hACE2 transgenic mice (*n* = 6), purchased from Jackson Laboratory (Bar Harbor, ME, USA) (genetic background (C57BL/6J × SJL/J)F2), were immunized by i.m. route with 40 μg of mRNA-RBD (G1: mNE-RBD, G2: mLNP-RBD or G3: LNP-1-RBD), with 1 × 10^7^ PFUs of MVA-RBD (G4) or with PBS (G5 and G6). At day 21 mice were boosted with 1 × 10^7^ PFUs of MVA-RBD (G1 to G4) or with PBS (G5 and G6) by i.m. route. Therefore, groups G1 to G3 were vaccinated with a heterologous mRNA/MVA vaccination regimen, while group G4 received two doses of the recombinant MVA-RBD vector (homologous MVA/MVA combination). Blood was harvested by submandibular bleeding at 20 days post-prime (d20) and 21 days post-boost (d42) for the analysis of SARS-CoV-2-specific BAbs and NAbs. At day 47, animals from G1 to G5 were challenged by intranasal (i.n.) route with a lethal dose (10^5^ PFUs) of SARS-CoV-2 (MAD6 strain) under isoflurane anesthesia. Animals from G6 remained unchallenged. Mice were monitored daily for weight, general health and survival for 14 days and those with more than a 25% of weight loss were euthanized. At the end of the study mice that survived were sacrificed, and lungs, nasal turbinates and serum samples were harvested and processed as previously described [33].

#### Ethics statement

The Ethical Committee of Animal Experimentation (CEEA) at CNB-CSIC and CISA-INIA and the Division of Animal Protection of the Comunidad de Madrid approved animal experiments using C57BL/6JOlaHsd and K18-hACE2 transgenic mice with permit number PROEX 169.4/20 and 161.5/20. All procedures adhered to EU guidelines and Spanish law under Royal Decree RD 53/2013.

#### Determination of SARS-CoV-2-Specific BAbs by enzyme-linked immunosorbent assay (ELISA) and NAbs by microneutralization test (MNT) assay

The presence of BAbs against SARS-CoV-2 RBD protein in serum was determined by ELISA as previously reported [33] using 2 μg/mL of recombinant SARS-CoV-2 RBD purified protein (kindly provided by Dr. José M: Casasnovas, CNB-CSIC, Madrid, Spain). Total IgG BAbs titers against RBD protein were determined as the last serum dilution that gave three times the mean optical density at 450 nm (OD_450_) of the control group (endpoint titer).

The capacity of the mouse sera to neutralize live SARS-CoV-2 virus (MAD6 isolate or different VoCs) was determined using a MNT assay performed in a BSL-3 laboratory at the CNB-CSIC as previously described [34]. To obtain the neutralization titers, half maximal inhibitory concentration (IC_50_) and 95% confidence intervals (95% CI) were calculated using a nonlinear regression model fit with settings for log agonist versus normalized response curve using GraphPad Prism 10.1.0 Software (GraphPad Software, San Diego, CA, USA).

#### mFcγRIV ADCC Reporter Bioassay

Antibodies mediating ADCC in mouse plasma were evaluated using a commercial mFcγRIV ADCC reporter bioassay kit (Promega, Madison, WI, USA) with some modifications. 96-well plates were coated with 3 μg/mL of trimeric-RBD overnight at 4°C, then washed and blocked with 5% bovine serum albumin. Serially 4-fold diluted pooled sera were added and incubated for 2 h at 37°C. Subsequently, 75.000 ADCC effector cells were added per well and incubated overnight at 37°C. Luminescence activity was measured using Bio-Glo luciferase reagent and a luminescence plate reader (Thermo Fisher). RBD-specific ADCC activity was reported as relative luminescence units (RLUs) calculated as follows: Fold induction = RLU of induced / RLU of no serum control (and relative to the maximum value obtained from sera of animals vaccinated with two doses of 5 μg of BNT162b2 human vaccine). Data were fitted to a 4PL curve to determine the half-maximal effective concentration (EC_50_) using GraphPad Prism 10.1.0 Software (GraphPad Software).

#### Analysis of the SARS-CoV-2-Specific T cell responses by ICS assay

To analyze the magnitude and phenotype of the SARS-CoV-2-specific T cells, 4 × 10^6^ splenocytes (erythrocyte-depleted)/well were seeded in 96-well plates and stimulated *ex vivo* for 6 h in complete Roswell Park Memorial Institute (RPMI; Sigma-Aldrich) 1640 medium (2 mM L-glutamine, 100 U/mL penicillin /100 μg/mL streptomycin, 10 mM Hepes and 0.01 mM β-mercaptoethanol) with 10% FBS (Sigma-Aldrich), monensin 1X (Invitrogen), 1 μL/mL Golgiplug (BD Biosciences, San Jose, CA, USA), anti-CD107a-FITC (BD Biosciences) and 2 μg/mL of a SARS-CoV-2 RBD peptide pool (PepMixTM SARS-CoV-2 (S-RBD), 53 peptides; JPT Peptide Technologies GmbH, Berlin, Germany). Unstimulated samples (RPMI) were used as control. After stimulation period, cells were washed, incubated with surface markers, permeabilized (Cytofix/Cytoperm kit; BD Biosciences) and stained intracellularly. The LIVE/DEAD Fixable Violet Dead Cell Stain Kit (Invitrogen) was used to exclude dead cells. For the analysis of SARS-CoV-2-specific T cells, the following fluorochrome-conjugated antibodies were used: CD3-PE-CF594, CD4-APC-Cy7 and CD8-V500 for phenotypic analyses, and CD107a-FITC, IL-2-APC, IFN-γ-PE-Cy7 and TNF-α-PE for functional analyses. All antibodies were from BD Biosciences. Cells were acquired in a GALLIOS flow cytometer (Beckman Coulter, Brea, CA, USA) and data analyses were carried out using FlowJo software (Version 10.4.2; Tree Star, Ashland, OR, USA). Lymphocyte-gated events ranged between 10^5^ and 5 × 10^5^. After gating, boolean combinations of single functional gates were generated to quantify the frequency of each response based on all the possible combinations of cytokine expression or differentiation markers. Background responses in the unstimulated controls (RPMI) were subtracted from those obtained in stimulated samples for each specific functional combination.

#### Analysis of SARS-CoV-2 RNA and cytokines by quantitative RT–PCR (RT-qPCR)

Lungs from transgenic K18-hACE2 mice were homogenized using a gentleMACS dissociator (Miltenyi Biotec, Bergisch Gladbach, Germany) in 2 mL of RLT buffer (Qiagen, Hilden, Germany) supplemented with β-mercaptoethanol (Sigma-Aldrich). Total RNA was isolated as previously described [33], and viral *RdRp* and *N* RNA copy numbers (copies/µL) were measured using the SARS-CoV-2 One-Step RT–PCR Kit II, RdRp and N genes, IVD (NZYTech, Lisboa, Portugal), following the manufacturer´s recommendations. mRNA expression levels of specific cytokine/chemokine genes *(Ifnβ-1, Il-10, Ip-10, Ccl-2, Il-24, Il-6, Il-12β, Timp1, Cxcl5 and Tnfα*) were analyzed using Taqman probes from Thermo Fisher Scientific [26]. Real-time PCR was conducted using a 7500 real-time PCR system from Applied Biosystems (Waltham, MA, USA). Data analysis was performed with 7500 software v2.0.6. Viral RNA levels were shown as copies/µL while cellular genes were expressed as relative RNA arbitrary units (A.U.) and were determined using the 2^−ΔΔCt^ method. All samples were referenced to a negative control group (PBS-uninfected mice) and cellular 28S rRNA was used for normalization. All samples were tested in duplicate. The primers, probes and the One-Step RT–PCR kit used for the detection of the different RNAs are listed in Supplementary Table 1.

#### Analysis of SARS-CoV-2 virus yields by plaque assay

Lung and nasal turbinate samples from K18-hACE2 mice were processed and analyzed for SARS-CoV-2 infectious virus using a plaque assay previously described [26]. Briefly, undiluted and serial 10-fold dilutions of homogenized lung tissue or nasal turbinate samples were added in triplicate to Vero-E6 cell monolayers seeded in 12-well plates at 5 × 10^5^ cells/well. After 1 h of adsorption, the inoculum was removed and the plates were incubated at 37°C with 5% CO_2_ in 2:1 DMEM 2X-4% FBS: Avicel® RC-591 (microcrystalline cellulose and carboxymethylcellulose sodium, DuPont Nutrition Biosciences ApS, Kongens Lyngby, Denmark). After 4 days, cells were fixed for 1 h with 10% formaldehyde (Sigma-Aldrich), the supernatant was removed and plaques were visualized by adding 0.5% crystal violet (Sigma-Aldrich). SARS-CoV-2 titers were determined as PFUs per gram of lung tissue or per milliliter of nasal turbinate.

#### Data analysis and statistics

For the statistical analysis of BAbs levels, the comparison between groups and time-points was performed by an ordinary one-way ANOVA and non-parametric test. NAbs titers and viral yields data were analyzed by an ordinary one-way ANOVA of transformed data followed by Tukey´s multiple comparison test. For the statistical analysis of ICS data, an approach that adjusts the values for the non-stimulated controls (RPMI) and calculates the confidence intervals and *p* values was used [35]. Only antigen responses significantly higher than the corresponding RPMI values were represented. All values are background-subtracted. For the analysis of weight loss data, we normalized the weight of each animal to 100% on day 0 and then measured the percentage variation from this baseline. A two-way ANOVA was performed with day and vaccination group as factors, including their interaction. For the statistical analysis of RT-qPCR data, unpaired non-parametric t test with Welch´s correction was used to establish differences between groups. Graphs and statistical analyses were performed using GraphPad Prism 10.1.0 software (GraphPad Software). The statistical significances are indicated as follows: *, *p *< 0.05; **, *p* < 0.005; ***, *p* < 0.001.

## Results

### Generation and in vitro characterization of MVA-RBD vaccine candidate expressing the soluble trimeric-RBD domain of the SARS-CoV-2 S protein

**Figure 1.**
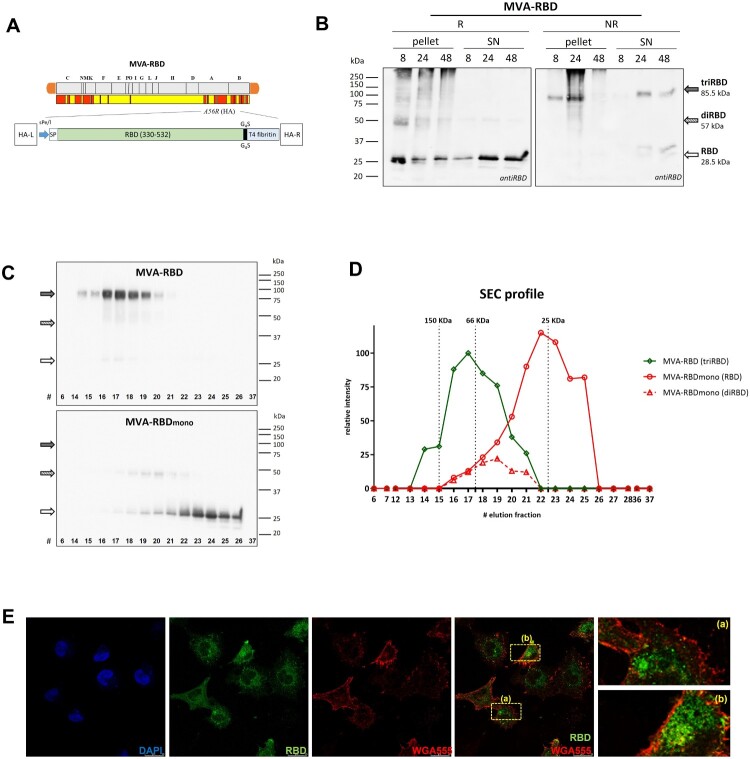
MVA-RBD recombinant virus efficiently expresses the trimeric-RBD of SARS-CoV-2. (A) Schematic representation of MVA-RBD virus genome generated by the insertion of *RBD* gene into the viral HA locus. (B) Time-course expression of RBD protein in HeLa cells infected with MVA-RBD. The expression of RBD protein was analyzed by WB in cellular pellets (pellet) and supernatants (SN) under reducing (R) and non-reducing (NR) conditions using an anti-RBD antibody. (C, D) SEC of RBD. Supernatants of cells infected with MVA-RBD or MVA-RBDmono were processed and analyzed by SEC as described in M&M. WB of the collected fractions is show in C, and the SEC profile resulting from the quantified amount of protein in each fraction is shown in D. Size exclusion markers in the SEC are indicated by dashed lines: Alcohol dehydrogenase-150 kDa, bovine albumin-66 kDa, and a monomeric RBD protein-27 kDa. (E) Subcellular localization of RBD protein by confocal microscopy.

In a previous study we designed and produced NE- and LNP-based prototypes encapsulating an mRNA encoding the soluble trimeric-RBD form of SARS-CoV-2 S protein [26]. In this work, we generated and characterized an MVA-based recombinant virus expressing the same RBD construct. The correct insertion of the *RBD* gene into the HA locus of MVA-WT (Figure 1A) was confirmed by PCR, with a specific 1392 bp product, indicating successful integration, and no parental contamination (Supplementary Figure 2A). DNA sequencing further confirmed the fidelity of the RBD sequence. The correct expression and secretion of the RBD protein was confirmed by WB analysis of cell extracts and supernatants obtained from HeLa cells infected with MVA-RBD. Trimeric-RBD form was observed under non-reducing conditions, reaching the maximum detection in the supernatant of infected cells at 24 h post-infection (Figure 1B). The trimeric conformation of the RBD protein was also verified by SEC of supernatants from HeLa cells infected with MVA-RBD or MVA-RBDmono. The trimeric-RBD protein (∼85.5 kDa) expressed from MVA-RBD and the monomeric RBD protein (∼28.5 kDa) expressed from MVA-RBDmono were detected by WB analysis of the collected fractions under non-reducing conditions (Figure 1C). The trimeric-RBD eluted from the column with a retention volume corresponding to a 70 kDa protein, whereas the monomeric-RBD retention volume was higher and similar to that of a monomeric-RBD protein (Figure 1D). The absence of RBD protein in the SEC fractions 23–24 collected with the trimeric sample indicated that no trimer dissociation occurred. Immunofluorescence analysis of HeLa cells infected with MVA-RBD revealed a diffuse and intense cytoplasmic distribution of the RBD protein (Figure 1E). Additionally, the analysis of RBD expression by WB after serial low MOI passages, along with the PCR data, confirmed its identity and demonstrate that MVA-RBD recombinant vector is highly stable (Supplementary Figures 2B and C).

### MVA boost enhanced RBD-Specific IgG and SARS-CoV-2 NAbs and induced RBD-Specific T cellular immune responses in vaccinated mice

**Figure 2.**
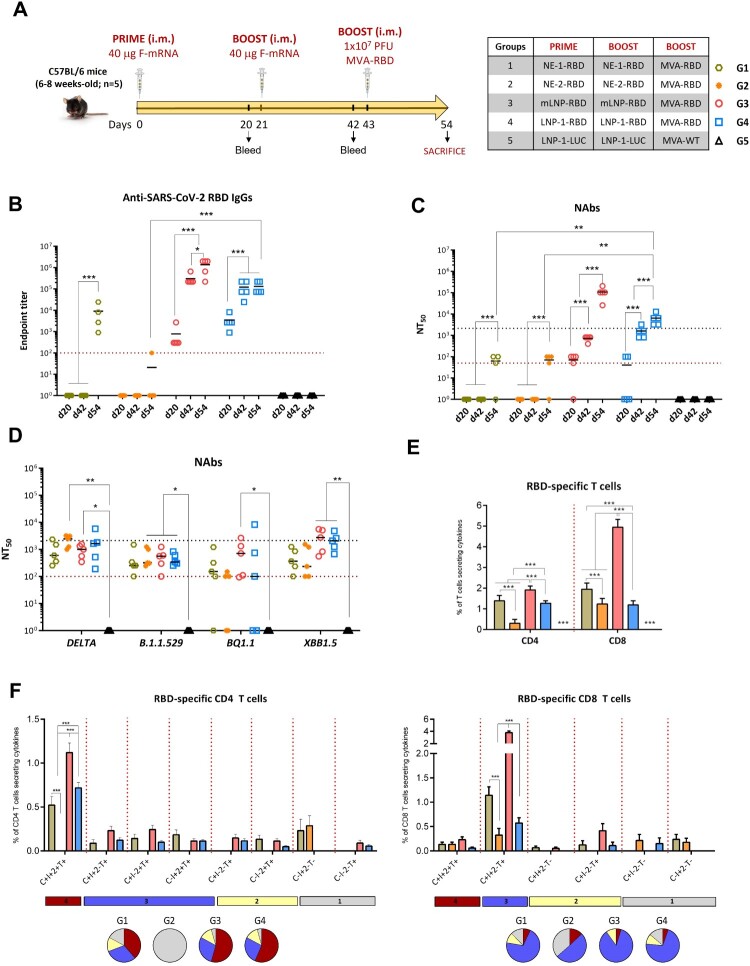
mRNA/MVA-RBD vaccination regimen enhances RBD-specific IgG BAbs and NAbs and elicits polyfunctional T cell immune responses in C57BL/6 mice. (A) Immunization schedule (as described in M&M). (B) Analysis of RBD-specific IgGs in individual serum samples at d20, d42 and d54. Data are shown as colored forms for each animal with geometric mean and SD. The red dotted line represents the lower limit of detection (LLD) of the assay. (C) Analysis of NAbs in individual serum samples at d20, d42 and d54. Upper black dotted line represents the levels obtained with the NIBSC 20/136 international standard plasma (pooled from 11 individuals recovered from SARS-CoV-2 infection). Bottom red dotted line represents the LLD of the assay. (D) NAbs against SARS-CoV-2 VoCs at d54. (E) Magnitude of SARS-CoV-2 RBD-specific CD4 or CD8 T cells at d11 post-MVA-RBD boost. The overall response represents the sum of the percentages of RBD-specific CD4 or CD8 T cells expressing CD107a and/or secreting IFN-γ and/or IL-2 and/or TNF-α. Data are background-subtracted. (F) Polyfunctional profile of SARS-CoV-2 RBD-specific CD4 (left) or CD8 (right) T cells. The positive combinations of the responses are indicated on the *x* axis, while the percentages of the functionally different cell populations within the total CD4 or CD8 T cells are represented on the *y* axis. Specific responses are grouped and color-coded based on the number of functions. C: CD107a; I: IFN-γ; 2: IL-2; T: TNF-α.

It is well-established that heterologous prime/boost combinations trigger more effectively broad T cell and antibody responses compared to homologous regimens [22,23,25]. Our previous research demonstrated that the mRNA/MVA combination expressing the same HIV-1 immunogens markedly enhanced the HIV-1-specfic T cell responses [24]. Based on these findings, we employed a similar heterologous regimen to characterize the SARS-CoV-2-specific immune response elicited by mRNA/MVA combination (Figure 2A).

In C57BL/6 mice, two doses of formulated mRNA-RBD followed by an MVA-RBD boost successfully induced RBD-specific BAbs, NAbs and polyfunctional T cell immune responses (Figure 2). The LNP-based mRNA vectors (mLNP-RBD and LNP-1-RBD) were more effective in priming humoral responses than the NE-based formulations (NE-1 and NE-2). After the second mRNA-RBD dose, all animals vaccinated with LNP prototypes elicited significant RBD-specific BAbs (titers ranging between 10^4^-10^5^) and NAbs (10^3^). In contrast, no detectable humoral responses were observed at this time-point in animals receiving NE-1-RBD or NE-2-RBD. Notably, the magnitude and frequency of both BAb and NAb responses were significantly enhanced after the MVA-RBD boost in all groups (Figures 2B and C), and were significantly higher in the group primed with LNP-based mRNA vectors (G2 and G3). After MVA-RBD boost (d54), and despite the variation observed between animals, we detected moderate levels of cross-reactive NAbs against Delta (B.167.2) and Omicron VoCs in all groups, with higher levels in mice immunized with LNP-based mRNA vectors, particularly against Omicron subvariants BQ.1.1 and XBB.1.5, where NT_50_ titers ranged from 10^3^ to 10^4^ in some animals (Figure 2D).

In terms of cellular responses, the mLNP-RBD/MVA-RBD regimen induced the highest RBD-specific CD4 and CD8 T cell responses, with predominance of the CD8 T cell compartment, followed by the NE-1-RBD/MVA-RBD combination (Figure 2E). The increase in SARS-CoV-2-specfic T cells observed in mLNP-RBD/MVA-RBD-immunized mice is likely due to the formulation, which includes C12-200, DOPE, cholesterol and Tween 80. These components, especially the combination of DOPE and cholesterol, are known to enhance the delivery and presentation of mRNA, leading to a more robust activation of both CD4 and CD8 T cells [36].

Analysis of IFN-γ, IL-2 and TNF-α production and CD107a expression identified eight distinct subsets of RBD-specific CD4 T cells and six subsets of CD8 T cell populations (Figure 2F). The CD4 T cell responses were highly polyfunctional in all groups, except in G2 (NE-2-RBD), with over 75% of CD4 T cells displaying multiple functions. The most representative population was CD4 T cells co-expressing CD107a + IFN-γ + IL-2 + TNF-α. Similarly, the CD8 T cell responses were highly polyfunctional in all groups, with more than 60% of CD8 T cells exhibiting multiple functions, especially CD107a + IFN-γ + TNF-α.

In summary, two doses of mLNP-RBD or LNP-1-RBD followed by an MVA-RBD boost significantly induced SARS-CoV-2-specific humoral immune responses and robust polyfunctional RBD-specific CD4 and CD8 T cell activation. This approach also increased BAbs and NAbs levels in NE-1- and NE-2-primed groups, which initially showed no humoral response, and expanded a balanced and polyfunctional T cell response generated by NE-based mRNA vectors.

### Efficacy study against SARS-CoV-2 of heterologous mRNA/MVA vs. homologous MVA/MVA vaccination regimens in susceptible K18-hACE2 mice

**Figure 3.**
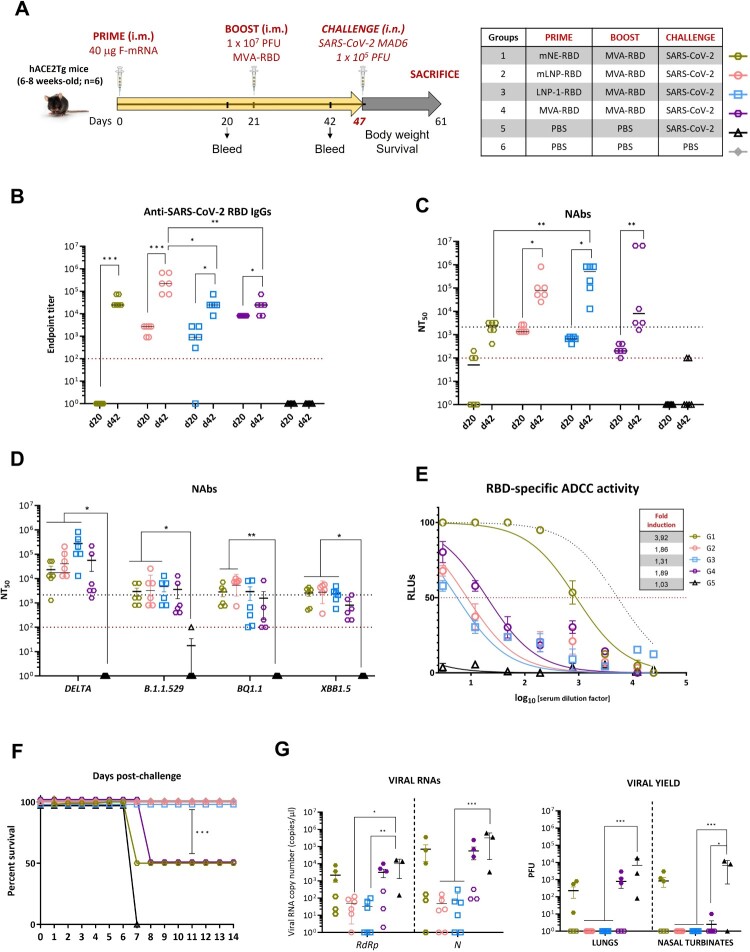
mRNA-LNP/MVA vaccination regimen elicits the highest levels of RBD-specific IgGs and NAbs and fully protects K18-hACE2 transgenic mice. (A) Immunization schedule (as described in M&M). (B) Analysis of RBD-specific IgGs in individual serum samples obtained at d20 and d42. The red dotted line represents the LLD of the assay. (C) Analysis of NAbs in individual serum samples at d20 and d42. Upper black dotted line represents the NIBSC 20/136 international standard plasma. Bottom red dotted line represented the LLD of the assay. (D) NAbs against VoCs at d42. (E) ADCC activity against SARS-CoV-2 trimeric-RBD protein in sera collected from vaccinated mice. Black dotted curve represents the levels obtained with the BNT162b2 mRNA vaccine. Red dotted line indicates the EC_50_. (F) Monitoring of mortality during 14 days after the SARS-CoV-2 challenge. Mice that lost more than 25% of the initial body weight were euthanized. (G) Left panel: Viral copy number of genomic (*RdRp*) and subgenomic (*N*) SARS-CoV-2 RNAs detected by one step RT-qPCR in lungs from individual mice at d7 p.c. (G5 and 50% of G1 and G4) or d14 p.c. (G2, G3 and 50% of G1 and G4). Mean RNA levels (in copies/µL) and SEM from duplicates of each lung sample are represented. Relative values were referred to PBS-challenged mice (G5). The filled shapes of G1 and G4 correspond to non-protected mice. Right panel: SARS-CoV-2 infectious virus in lungs and nasal turbinates. Mean PFU (PFUs/g of lung tissue or PFUs/mL of nasal turbinates) and SEM from triplicates of each sample are represented. The filled shapes of G1 and G4 correspond to non-protected mice.

To evaluate the protective potential of heterologous mRNA/MVA versus homologous MVA/MVA vaccination regimens against SARS-CoV-2, we conducted an efficacy study in susceptible K18-hACE2 transgenic mice ­following the schedule depicted in Figure 3A.

#### Analysis of the SARS-CoV-2-specific humoral immune responses at pre-challenge

We characterized the SARS-CoV-2-specific humoral response induced by mNE-RBD, mLNP-RBD, LNP-1-RBD and MVA-RBD at 20 days post-prime (d20) and 21 days post-MVA-RBD boost (d42). As shown in Figure 3B, at d20 mice immunized with a single dose of mLNP-RBD, LNP-1-RBD or MVA-RBD exhibited RBD-specific BAb titers exceeding 10^3^, which further increased at d42 reaching endpoint titers up to 10^5^. In the mNE-RBD-immunized group RBD-specific BAbs were undetectable at d20 but reached titers ranging between 10^4^–10^5^ after MVA-RBD boost. Similar results were observed when we analyzed the neutralization capacity of the sera against SARS-CoV-2 virus (Figure 3C), with one dose of mLNP-RBD or LNP-1-RBD inducing SARS-CoV-2-specific NAbs at d20 that were boosted after the MVA-RBD dose at d42, achieving NT_50_ titers of 10^4^-10^6^. The mNE-RBD group only showed NAbs after MVA-RBD boost, with titers of 10^3^-10^4^, which were similar to the titers obtained after MVA-RBD/MVA-RBD regimen. In addition, and despite the variation detected between animals, serum from the four groups at d42 effectively cross-neutralized different SARS-CoV-2 VoCs with NT_50_ values of 10^4^–10^6^ for Delta VoC, and 10^3^–10^4^ for Omicron lineage (Figure 3D), with heterologous mRNA/MVA regimen exhibiting the highest levels of cross-reactive NAbs. We also evaluated the RBD-specific ADCC activity of sera from immunized mice. As shown in Figure 3E, mNE-RBD induced the highest ADCC activity, reaching nearly a 4-fold increase compared to LNP-RBD-based vectors and the MVA/MVA vaccinated group, which showed inductions ranging between 1.3- and 1.9-fold.

#### Protection from mortality and SARS-CoV-2 virus replication induced by mRNA/MVA vaccination regimen

Next, we compared the protective efficacy of mRNA/MVA vs. MVA/MVA vaccination regimens in susceptible mice after SARS-CoV-2 live virus challenge. As shown in Figure 3F, all non-challenged mice treated with PBS (G6) survived, whereas PBS-challenged mice (G5) were sacrificed 7 days post-challenge (d7 p.c.) because their body weight loss exceeded the ethically permissible limit. Among the groups exposed to the SARS-CoV-2 virus, 50% of the mice inoculated with mNE-RBD/MVA-RBD (G1) or MVA-RBD/MVA-RBD regimens (G4) survived. In contrast, all mice vaccinated with mLNP-RBD/MVA-RBD or LNP-1-RBD/MVA-RBD regimens (G2 and G3) survived until the end of the study (d14 p.c.) (Figure 3F). Analysis of SARS-CoV-2 virus replication in lung samples revealed a significant reduction in SARS-CoV-2 replication (copy number of genomic *RdRp* and subgenomic *N* mRNAs) in all surviving LNP-immunized animals compared to PBS-challenged control group (G5) despite variations between animals (Figure 3G, left panel). This reduction correlated with the analysis of viral yields in lung homogenates and nasal turbinates, where no virus replication was detected in mice immunized with mLNP-RBD or LNP-1-RBD compared to PBS-challenged control mice, whereas only partial protection was observed in half of the animals of G1 and G4 (Figure 3G, right panel). These results demonstrate that the LNP-mRNA/MVA vaccination regimen effectively controlled SARS-CoV-2 lethality and virus replication in a susceptible mouse model.

### mLNP-RBD and LNP-1-RBD/MVA vaccination regimen differentially regulated the pro-inflammatory cytokine and chemokine profiles in lungs from challenged K18-hACE2 mice

**Figure 4.**
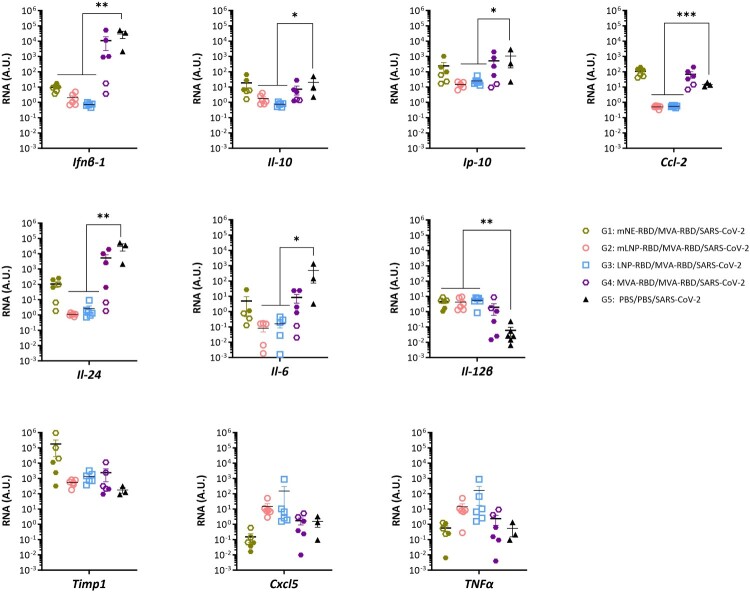
Cytokine and chemokine profile in lungs from vaccinated K18-hACE2 transgenic mice after SARS-CoV-2 challenge. Detection of RNA levels of pro-inflammatory cytokines and chemokines RNA by one step RT-qPCR at d7 p.c. for non-protected mice (G5 and 50% of G1 and G4, filled shapes) or at d14 p.c. for protected mice (G2, G3 and 50% of G1 and G4, empty shapes). Values were normalized to cellular 28S. Mean RNA levels (in A.U.) and SD from duplicates for each animal were related to control animal mock.

Given the association of pro-inflammatory cytokine up-regulation with COVID-19 disease progression and severity often referred to as a cytokine storm [37], we compared by RT-qPCR in lung homogenates from challenged mice the effect of heterologous mRNA/MVA vs. homologous MVA/MVA immunization regimens on cytokine expression pattern. As shown in Figure 4, both the mLNP-RBD- and LNP-1-RBD-primed groups exhibited significant and similar patterns of gene regulation. Notably, there was a down-regulation in the mRNA levels of *Ifnβ-1, Il-10*, *Cxcl10* (*Ip-10*), *Ccl-2, Il-24* and *Il-6* at d14 p.c. compared to the PBS-challenged group at d7 p.c. On the other hand, *Il-12β* was significantly up-regulated in all heterologous mRNA/MVA vaccinated groups. Furthermore, although not statistically significant, an increased expression of *Timp1, Cxcl5 and Tnfα* genes were observed in both LNP-primed groups. These findings indicate that animals that elicited effective NAbs were also able to protect against the SARS-CoV-2 challenge and that heterologous mLNP-RBD/MVA-RBD and LNP-1-RBD/MVA-RBD vaccination regimens were the most effective mitigating the cytokine storm typically triggered by SARS-CoV-2 infection.

## Discussion

The emergence of SARS-CoV-2 has become a major public health threat, causing over 7 million deaths since 2020 [38]. Recent insights into the pathogenesis of the virus have informed the development of COVID-19 vaccines and therapeutic strategies [1,39]. However, questions still remain on the durability of the immune responses and the diminished ability of the current vaccines to control VoCs. In a previous study, we designed an mRNA vector encoding a soluble trimeric-RBD form that, when formulated using alternative nanocarriers (LNP, NE and NC) and tested in a homologous mRNA/mRNA regimen, activated robust RBD-specific immune responses. These responses provided protection against SARS-CoV-2 infection in susceptible K18-hACE2 transgenic mice comparable to that elicited by Pfizer-BioNTech mRNA vaccine encoding the full-length S protein [26], suggesting that targeting the RBD alone in its trimeric form can achieve an immunogenicity profile similar to that of the full-length S protein.

In an effort to adapt current vaccine protocols to flexible regimens, in this study we generated an MVA-based recombinant virus expressing the soluble trimeric-RBD (MVA-RBD) to be used in combination with the mRNA vaccines. The rationale for the heterologous mRNA/MVA combination lies in the ability of MVA-based vectors to enhance the magnitude and durability of immune responses primed by various vaccine platforms (DNA, mRNA, protein subunits and different viral vectors) and to confer robust protection against infections [22–25,28]. Unlike other viral vectors, MVA does not integrate into the host genome, reducing the risk of insertional mutagenesis, and is safe for immunocompromised individuals [40], a significant advantage over some adenovirus vectors which can replicate in specific settings, potentially causing disease in these populations. Additionally, MVA vectors induce broad and durable immune responses by activating multiple pathways crucial for innate and adaptive immunity, making them particularly effective against complex viruses like SARS-CoV-2 [41]. This comprehensive immune activation is often more limited in other vaccine types, such as inactivated or recombinant vaccines, which primarily induce antibody responses.

It has been previously demonstrated that a prime/boost combination of self-amplifying mRNA vaccines with an adenovirus vaccine, both targeting the SARS-CoV-2 S protein, elicited stronger specific immune responses than the homologous combination. This included enhanced levels of both BAbs and NAbs, as well as T cell activation [42,43]. Moreover, different clinical trials have shown that such heterologous combinations, involving mRNA and adenovirus vectors expressing the S protein, significantly enhanced the immune responses to SARS-CoV-2 [44-46]. In the case of MVA vectors, preliminary data from clinical studies in humans indicated that boosting individuals who have already received two or more doses of an mRNA vaccine with an MVA vector expressing a prefusion-stabilized SARS-CoV-2 S protein was better compared to homologous regimen [47,48].

In our study, the induction in C57BL/6 mice of robust RBD-specific BAbs and cross-reactive NAbs against different SARS-CoV-2 VoCs, including Delta and Omicron, and the activation of potent and polyfunctional RBD-specific CD4 and CD8 T cell responses underscores the efficacy of the mRNA/MVA vaccination approach, with LNP-based mRNA vectors being more effective in priming humoral responses than the NE-based formulations. In efficacy studies using transgenic K18-hACE2 mice, groups primed with mLNP-RBD or LNP-1-RBD followed by MVA-RBD boost not only survived the SARS-CoV-2 challenge, but also exhibited significant reductions in viral replication and viral yield in the lungs and nasal turbinates compared to mNE-RBD/MVA-RBD and MVA-RBD/MVA-RBD groups, which showed a 50% survival rate. Although LNPs were superior to mNE in priming the humoral immune response, the mNE-RBD-primed group exhibited the highest score in terms of ADCC induction. ADCC is crucial in the defense against SARS-CoV-2 by complementing NAbs and contributing to the inflammatory response, which must be carefully regulated to avoid tissue damage [49,50]. However, the group with the highest ADCC values only achieves 50% protection, highlighting the complexity of an effective immune response. This indicates that while ADCC is important, it is insufficient alone to confer full protection. Comprehensive immunity against SARS-CoV-2 requires a balanced response that includes strong NAbs, effective T cell responses and well-regulated Fc-mediated effector functions. Hence, vaccines and treatments must aim to elicit this multi-dimensional immune response for optimal protection [51].

The efficacy observed in transgenic K18-hACE2 mice correlates with the cytokine and chemokine profiles identified in the lungs of protected mice. Notably, there was a significant reduction of *Ifnβ-1, Ip-10, Ccl-2 and Il-24* RNA levels. These proteins play critical roles in NFKB, JAK/STAT and TGFB pathways, which are known targets of SARS-CoV-2 miRNAs [37,52]. Additionally, the protected animals also exhibited a significant down-regulation in the RNA levels of other important inflammatory mediators such as *Il-6* and *Il-10*, which have been associated with COVID-19 disease progression and severity. Conversely, we detected a significant up-regulation of the *NK cell stimulatory factor* *Il-12β* and a trend towards an up-regulation of *Timp1, Cxcl5* and *Tnfα* RNA levels*.* This up-regulation could play a crucial role in the defense against the virus, as it has been demonstrated against bacteria [53], and in promoting infiltration and neutrophil recruitment to the site of infection [54]. The improved cytokine profile can be attributed to the way in which vaccines prime the immune system. mRNA vaccines primarily activate strong type I interferon and Th1 responses, promoting rapid antiviral action and robust adaptive immunity, while MVA vaccines stimulate a broader immune activation, including extensive engagement of dendritic cells and a mix of cytokines that support both immediate and long-term immune responses. Therefore, this mRNA/MVA regimen could synergistically optimize both immediate and sustained immune responses, enabling effective viral clearance and maintaining lung tissue integrity, with the potential to effectively adapt to diverse viral variants ensuring a durable and broad immune protection.

Overall, our study underscores the promising efficacy of a heterologous mRNA/MVA vaccination regimen against SARS-CoV-2, particularly with the use of alternative nanocarriers that circumvent intellectual property restrictions for mRNA formulation. The adaptability of this vaccine strategy to various formulations could be crucial for managing the current pandemic and preparing for future outbreaks, presenting a potent mRNA-RBD/MVA-RBD strategy to achieve broad and lasting COVID-19 immunity.

## Supplementary Material

supplementary table1.jpg

supplementary figure2.jpg

supplementary figure1.jpg
