# Supplementary figures and images for: Heterologous mRNA/MVA delivering trimeric-RBD as effective vaccination regimen against SARS-CoV-2: COVARNA Consortium
Source: Emerg Microbes Infect. 2024 Aug 1;13(1):2387906. doi: 10.1080/22221751.2024.2387906 (PMC11313003; doi:10.1080/22221751.2024.2387906)

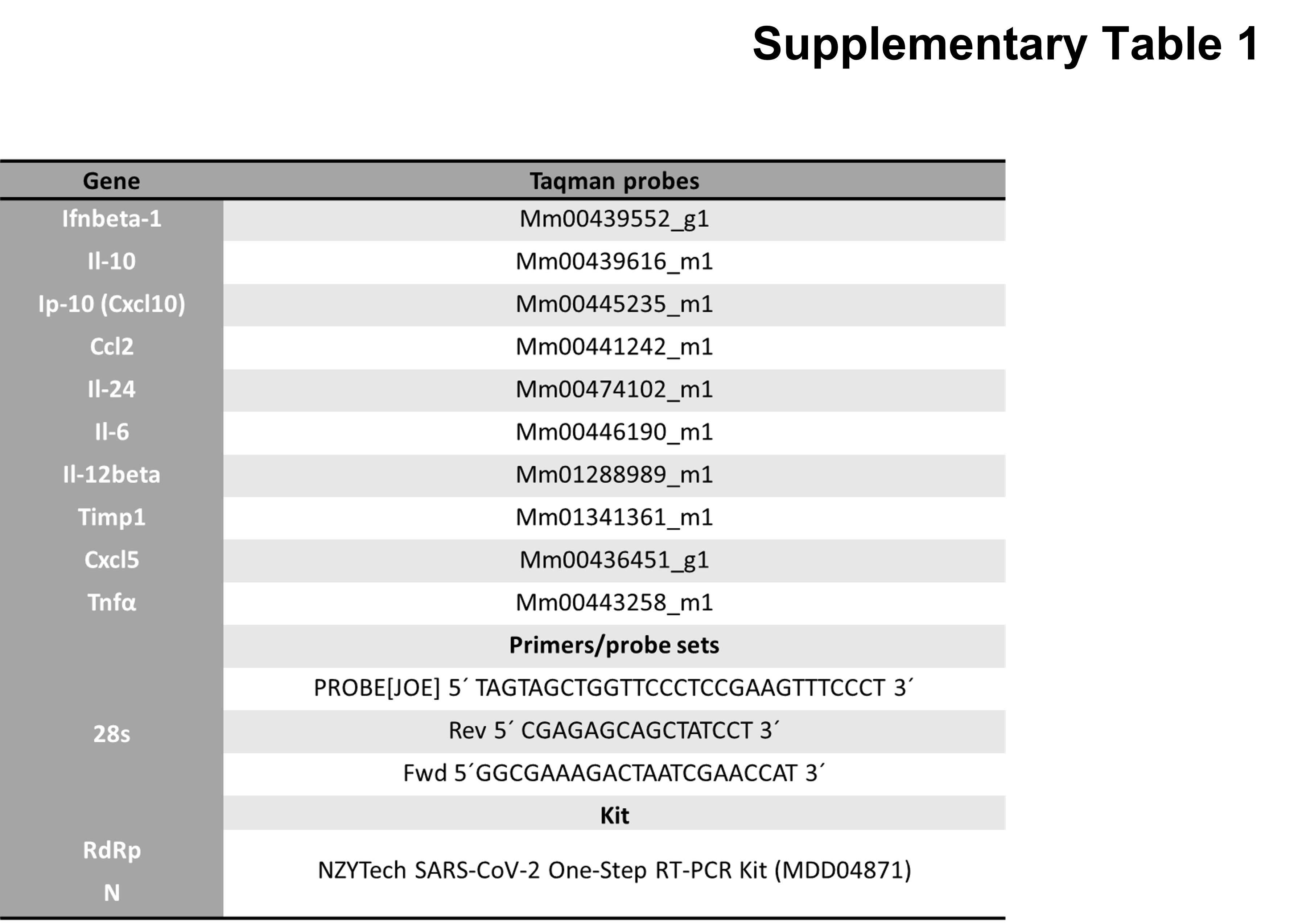

Supplement: supplementary table1.jpg [file TEMI_A_2387906_SM7308.jpg]

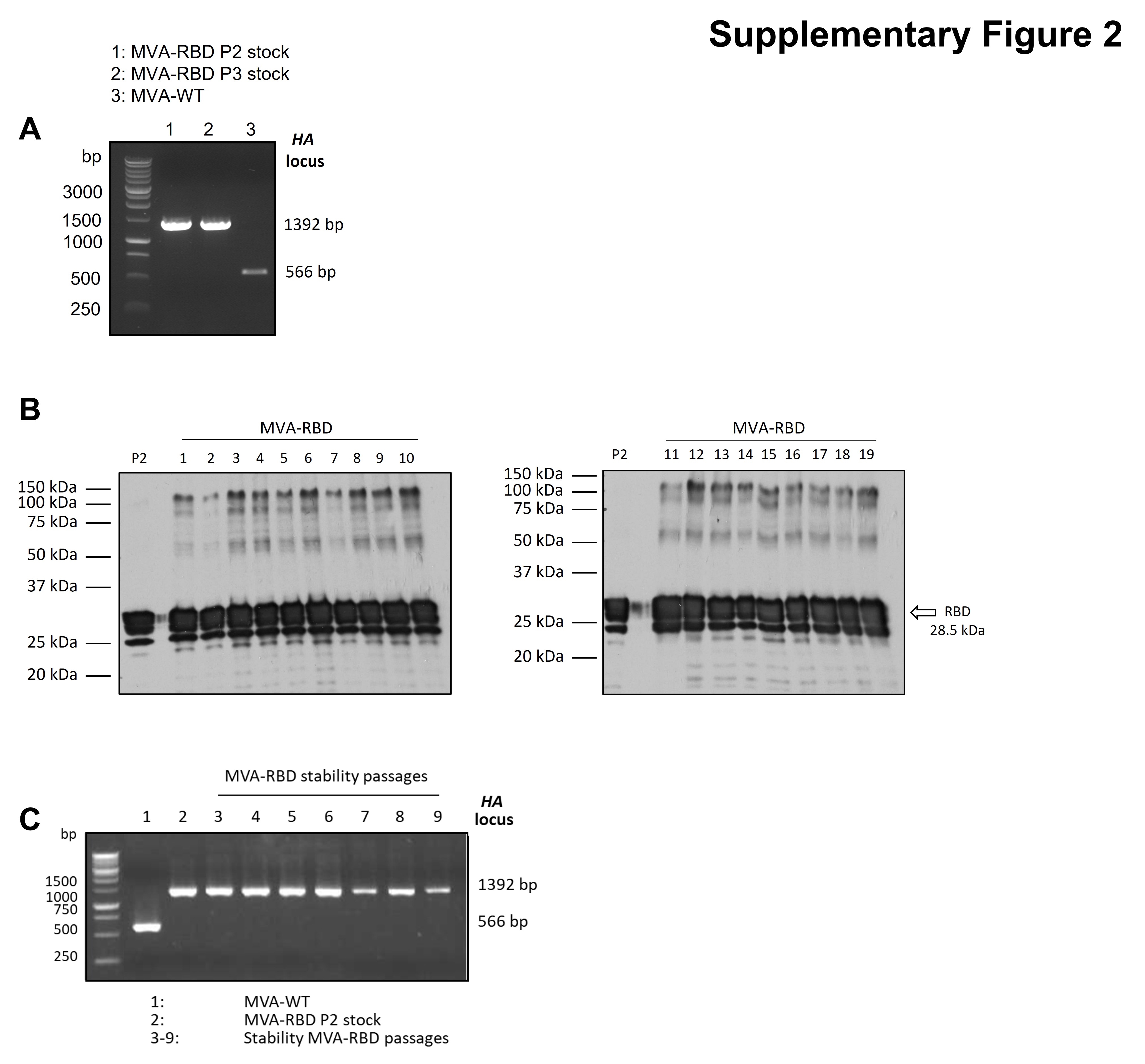

Supplement: supplementary figure2.jpg [file TEMI_A_2387906_SM7307.jpg]

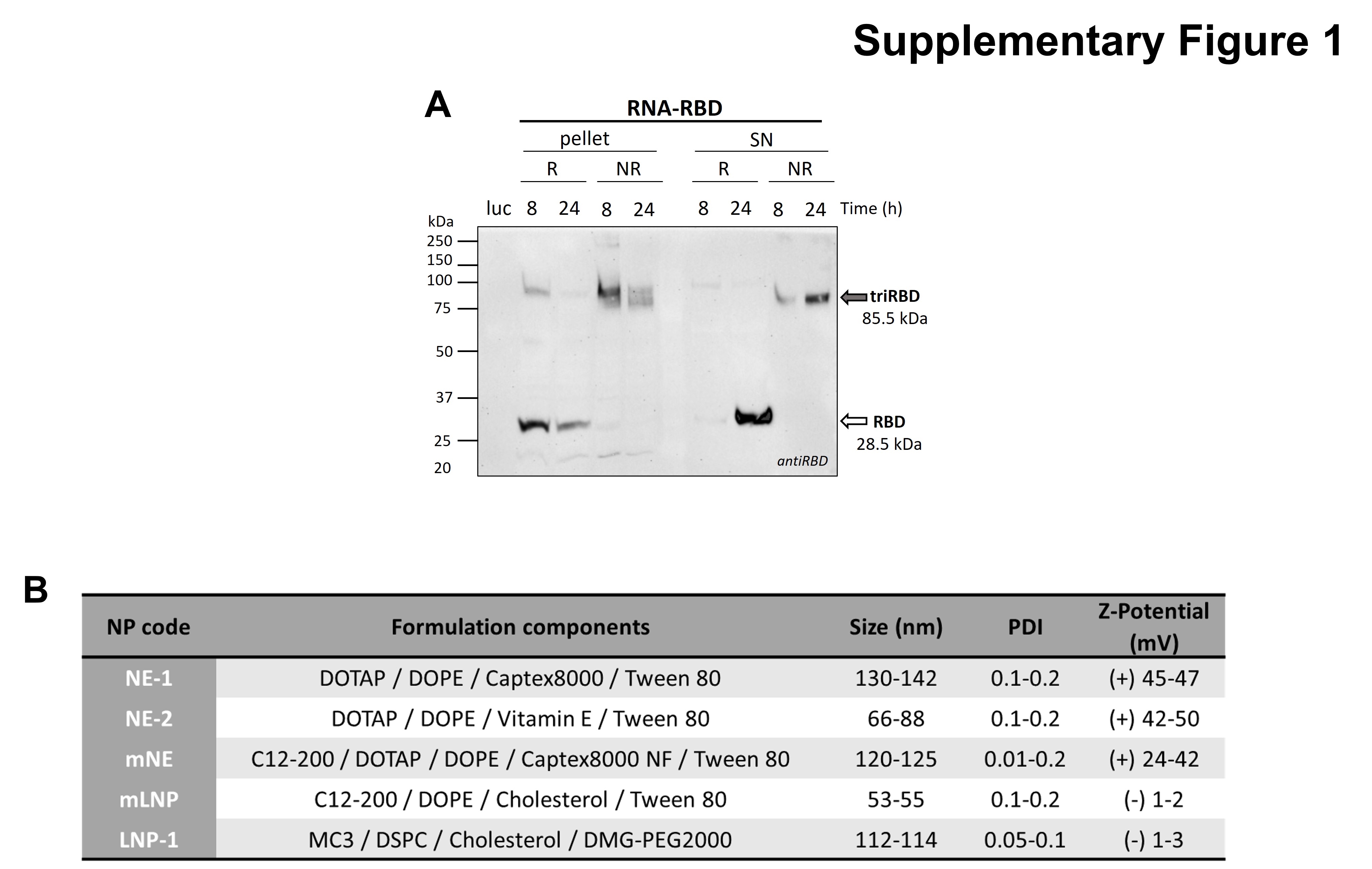

Supplement: supplementary figure1.jpg [file TEMI_A_2387906_SM7306.jpg]
